# Efficacy of gel immersion endoscopic ultrasonography for delineating the duodenal papilla and pancreatobiliary ducts: A retrospective study with video

**DOI:** 10.1002/deo2.158

**Published:** 2022-08-04

**Authors:** Haruka Toyonaga, Toshifumi Kin, Kosuke Iwano, Risa Nakamura, Takao Shimizu, Koki Chikugo, Tatsuya Ishii, Hiroshi Nasuno, Tsuyoshi Hayashi, Kuniyuki Takahashi, Hajime Yamazaki, Akio Katanuma

**Affiliations:** ^1^ Center for Gastroenterology Teine Keijinkai Hospital Hokkaido Japan; ^2^ Department of Community Medicine, Section of Clinical Epidemiology, Graduate School of Medicine Kyoto University Kyoto Japan

**Keywords:** duodenal papilla, endoscopic ultrasonography, gel immersion endoscopic ultrasonography, pancreatobiliary ducts, papilla of Vater

## Abstract

**Objectives:**

Endoscopic ultrasonography is an important examination for periampullary diseases. The duodenum is filled with water to ensure a clear image and distend the duodenal wall without burying the papilla within duodenal folds; however, peristalsis frequently makes it difficult to maintain water within the duodenum. The gel immersion method (intestine is filled with viscosity gel) has recently been attracting attention. We evaluated the usefulness of using this method for endoscopic ultrasonography to detect and delineate the major duodenal papilla.

**Methods:**

Fifty‐nine consecutive patients who underwent gel immersion‐endoscopic ultrasonography between February and March 2021 were included retrospectively. The papilla was observed by filling the duodenum with clear viscosity gel. Outcomes were the rate of duodenal distention, delineation rates of the papilla, the time required for delineation, volume of the gel used, and adverse events.

**Results:**

Duodenal distention was excellent, good, and poor in 58%, 34%, and 7% of cases, respectively. The delineation rates of the papilla in the axial and longitudinal views were 98% and 66%, respectively. The median time required to delineate the papilla in each view was 3.1 (range, 1.0–1.4) and 7.9 (1.9–28.6) min; the median volume of the gel used was 80 (30–150) ml and 100 (50–200) ml, respectively. No adverse events were noted.

**Conclusions:**

Gel immersion‐endoscopic ultrasonography provided sufficient duodenal distention, leading to high rates of detection and delineation of the papilla using a small volume of gel within a short time. This method may be useful for the evaluation of the ampullary region.

## INTRODUCTION

Endoscopic ultrasonography (EUS) is a useful examination for periampullary diseases, such as undefined causes of hepatopancreatobiliary enzyme elevation, pancreatobiliary duct dilation, and periampullary tumors.^1^ In deciding the appropriate therapeutic method for ampullary tumors, the evaluation of the tumor extension, such as intraductal spreading or depth of the invasion, is important.^2^, ^3^ For the surveillance of periampullary regions using EUS, the duodenum is usually filled with water to ensure a clear image and to distend the duodenal wall without burying the papilla within duodenal folds^4^; however, peristalsis frequently makes it difficult to maintain water within the duodenum over the necessary period of time.

Recently, the gel immersion (GI) method has been introduced for useful endoscopic hemostasis; viscosity gel (VISCOCLEAR; Otsuka Pharmaceutical Factory, Inc., Tokushima, Japan) remains in the intestinal lumen over a prolonged period of time and secures the endoscopic visual field without interrupting the blood flow.^5^ The evaluation of esophageal lesions using the GI method in EUS surveillance (GI‐EUS) has been reported.^6^, ^7^, ^8^ However, the efficacy of GI‐EUS in the pancreatobiliary regions has not been well examined. Thus, we conducted a study to determine the efficacy of GI‐EUS to detect the duodenal papilla and pancreatobiliary ducts.

## METHODS

### Participant identification and ethics statements

This retrospective observational study was conducted at the Center for Gastroenterology, Teine Keijinkai Hospital. We identified consecutive patients who underwent GI‐EUS from a prospectively collected EUS database between February and March 2021. This study was approved by the Teine Keijinkai Hospital Institutional Review Board (approval number: 2‐020408‐00; registration date: April 22, 2021). Informed consent for the study participation was obtained in the form of an opt‐out on the hospital's website.

### GI‐EUS procedures

All GI‐EUS examinations were conducted by experts, with ≥10 years of EUS experience and who had performed ≥2000 pancreatobiliary EUS examinations, or by trainees under the supervision of the experts. All procedures were performed using radial or curved linear echoendoscopes (GF‐UE260‐AL5, GF‐UCT260, or GF‐UE290; Olympus Co., Tokyo, Japan) with a dedicated ultrasound processor (EU‐ME2 Premier Plus; Olympus Co.).

EUS surveillance was conducted with the patient under conscious sedation (midazolam and diazepam) and antispasmodic drug administration (scopolamine butylbromide and glucagon) using the Tissue Harmonic Echo mode without contrast agents. After inserting the scope into the duodenal descending part using a short scope position, we filled the duodenal lumen with VISCOCLEAR to ensure a clear image and avoid compressing the papilla (Video 1). The gel was additionally injected in cases where it was difficult to maintain the duodenal lumen distention owing to the outflow of the gel. The balloon attached to the tip of the endosonoscope was either not used, or inflated to the degree that it would not squeeze the papilla. After the identification of the periampullary region as a hypoechoic area as compared with the surrounding pancreas, we tried to detect the papilla that presented as a protruded region with penetration of the intrapancreatic pancreatobiliary ducts. After detecting the papilla, we observed intrapapillary pancreatobiliary ducts running through the papilla and delineated the longitudinal view of the intrapancreatic pancreatobiliary ducts, duodenal muscular layer, and papilla. All procedures were digitally recorded as movies.

### Outcome measures

**FIGURE 1:**
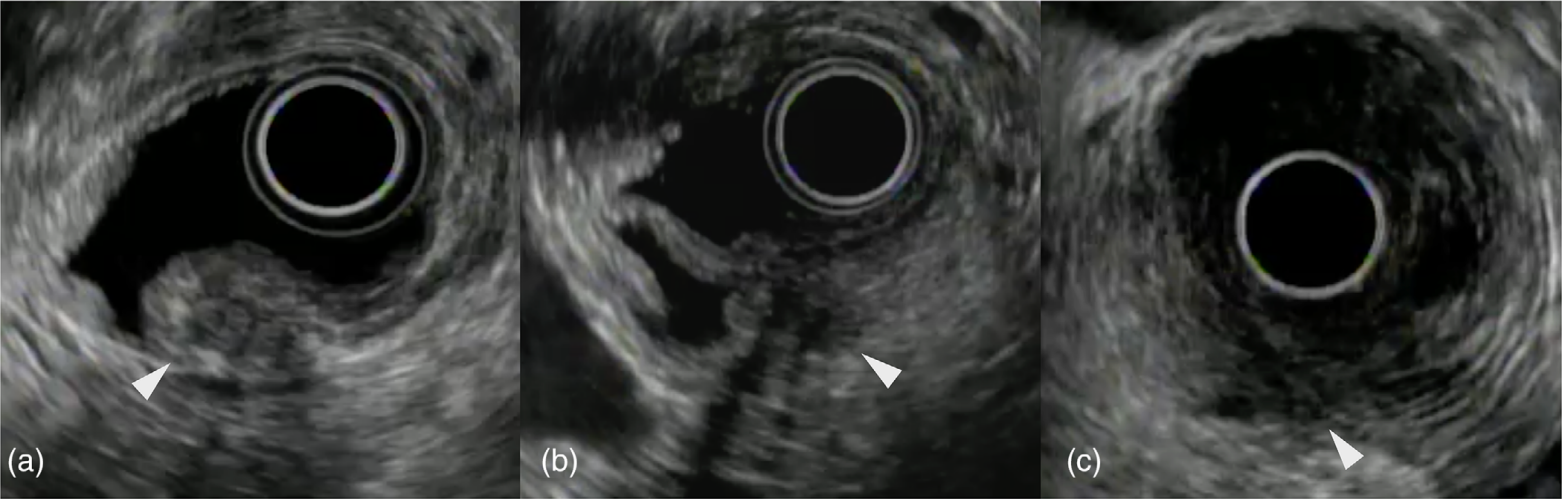
The effect of duodenal lumen distention with gel immersion is evaluated using a three‐grade scoring system. The arrowhead indicates the duodenal papilla. (a) Excellent: The duodenal lumen is well distended, and the papilla remains unburied in the duodenal fold. (b) Good: The duodenal lumen is distended, but the papilla is in contact with the mucosal folds. (c) Poor: The duodenal lumen is poorly distended, and the papilla is buried in the mucosal folds

The outcome measures were the degree of duodenal lumen distention, detection rates of the papilla and intrapapillary pancreatobiliary ducts, delineation rate of the papilla in the longitudinal view, the time required to detect the papilla and perform delineation, the volume of gel required, and adverse events associated with the procedure occurring within 3 days. The degree of duodenal lumen distention with GI was evaluated as follows: excellent, the duodenal lumen is distended enough that the papilla remains unburied in the duodenal fold; good, the duodenal lumen is distended, but the papilla is in contact with the mucosal folds; and poor, the duodenal lumen is not distended causing the papilla to be buried in the mucosal folds (Table 1 and Figure 1a–c).

**TABLE 1:** Definitions of outcomes

Effect of duodenal lumen distention	
Excellent	The duodenal lumen is well distended, and the papilla can remain unburied in the duodenal folds.
Good	The duodenal lumen is distended, but the papilla is in contact with the mucosal folds.
Poor	The duodenal lumen is poorly distended, and the papilla is buried in the mucosal folds.
Detection of the papilla	Visualization of the protruding region in the duodenum where the pancreatobiliary duct penetrates the duodenal muscular layer
Detection of the intrapapillary pancreatobiliary ducts	Ducts running through the papilla continuously from the pancreatobiliary duct in the pancreas
Delineation of the papilla in the longitudinal view	The papilla and pancreatobiliary duct penetrating the duodenal muscular layer are delineated simultaneously in the longitudinal view

**FIGURE 2:**
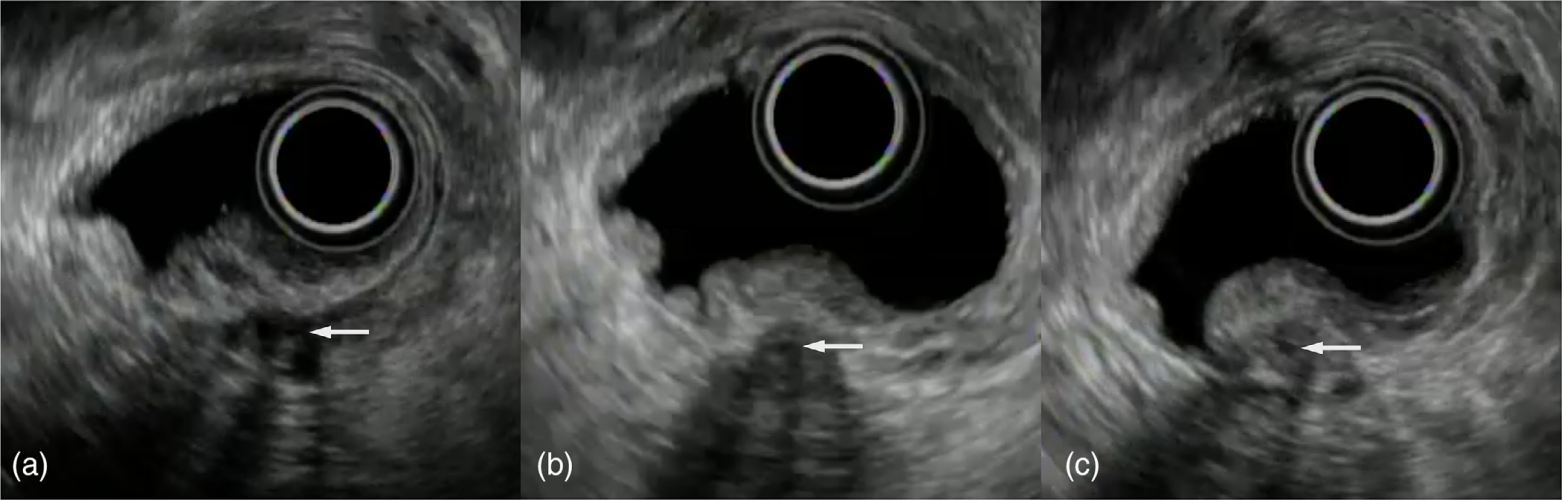
Detection of the duodenal papilla. (a) The intrapancreatic pancreatobiliary ducts (arrow) can be identified and (b) traced downstream to the site penetrating the duodenal muscular layer. (c) The papilla can be recognized as a protruding region on the duodenal lumen side. The intrapapillary pancreatobiliary ducts can be also detected (arrow)

**FIGURE 3:**
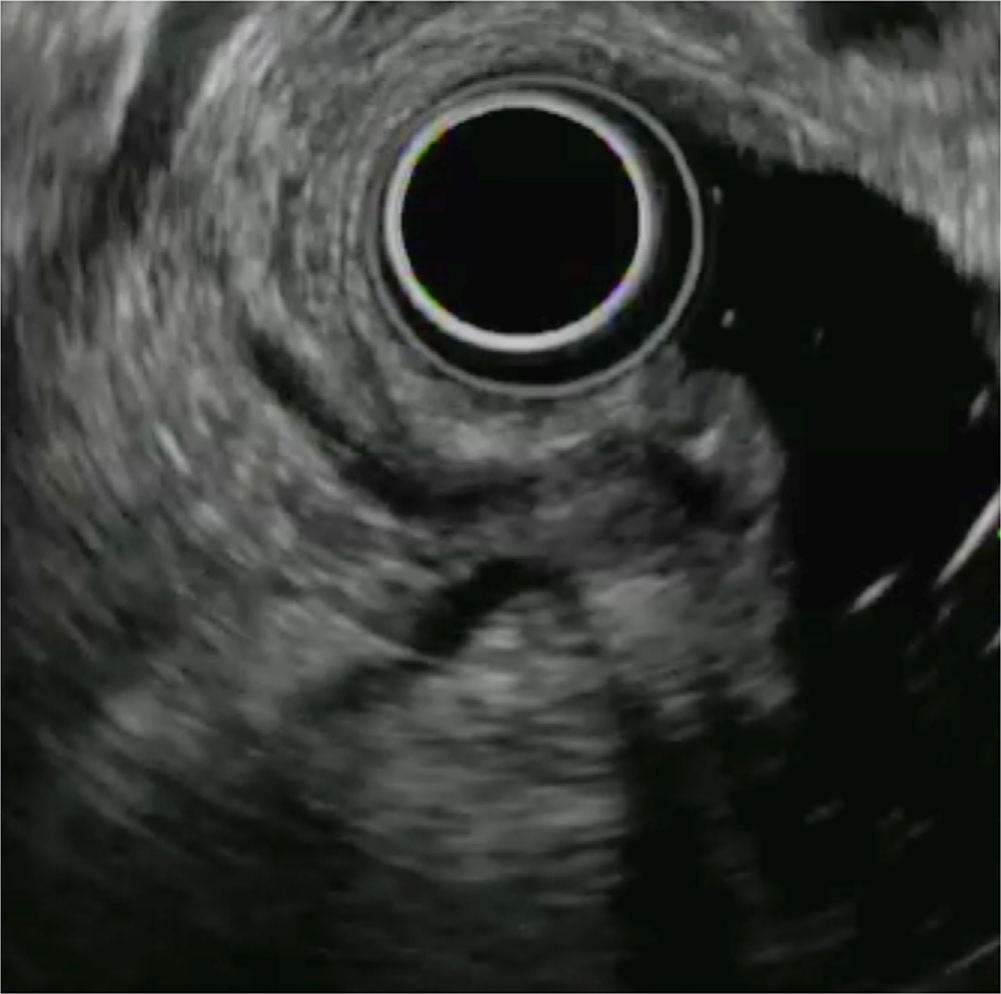
Delineation of the papilla in the longitudinal view. The papilla and pancreatobiliary ducts penetrating the duodenal muscular layer are delineated simultaneously in the longitudinal view

Successful detection of the papilla was defined as the identification of a protruding region in the duodenum, where the pancreatobiliary duct penetrated the duodenal muscular layer (Figure 2a). Detection of the intrapapillary pancreatobiliary duct was considered when the intrapancreatic pancreatobiliary ducts continuously ran through the papilla (Figure 2b,c). Delineation of the papilla in the longitudinal view was achieved by the simultaneous visualization of the papilla and pancreatobiliary duct penetrating the duodenal muscular layer in the longitudinal view (Figure 3).

The amount of gel used was estimated until successful delineation of the papilla in both the axial and longitudinal views was completed. The required time to detect the papilla was measured from the moment the scope was inserted into the duodenal descending portion until the delineation was completed or terminated.

The degree of duodenal lumen distention, successful detection of the papilla or intrapapillary pancreatobiliary ducts, successful delineation of the papilla in the longitudinal view, and the time required for detecting and delineating the papilla were evaluated by reviewing the stored recording of the procedures.

### Evaluations

The evaluation of outcomes was independently conducted by two pancreatobiliary EUS experts with ≥10 years of EUS experience in the pancreatobiliary field (Toshifumi Kin and Kuniyuki Takahashi), who were blinded to any information regarding the patient's data, findings from other imaging examinations, and clinical outcomes. In cases of discordance in their evaluations, another expert (Akio Katanuma) made the final assessment. Inter‐observer agreement was also examined to verify the validity of these evaluations.

### Data and statistical analyses

All continuous variables are presented as medians and ranges. All categorical variables are expressed as proportions and frequencies. Inter‐observer agreement was calculated to assess the concordance between the two observers using the first‐order agreement (AC1) statistics.^9^ The value of AC1 statistics was interpreted according to the standard classification: 0.00–0.20, slight agreement; 0.21–0.40, fair agreement; 0.41–0.60, moderate agreement; 0.61–0.80, substantial agreement; and 0.81–1.00, almost perfect agreement.^9^, ^10^ All statistical analyses were performed using EZR^11^ (version 1.54; Saitama Medical Center, Jichi Medical University, Saitama, Japan), a graphical user interface for R (version 4.1.1; R Foundation for Statistical Computing, Vienna, Austria).

## RESULTS

### Patient characteristics

During the study period, 78 consecutive patients who underwent pancreatobiliary EUS were investigated, as were 59 patients (76%) who underwent GI‐EUS (Table 2) for detection of the papilla. The remaining 19 patients did not receive GI‐EUS because they had lesions requiring close examination other than the papilla and there was insufficient time for observation of the normal papilla. Patients’ median age was 69 (range, 48–87) years, and the male‐to‐female ratio was 24:35. Previous surgical treatment was noted in six patients, including one patient who underwent distal gastrectomy with Billroth I reconstruction.

**TABLE 2:** Patients’ characteristics (*n* = 59)

Age, median (range), years	69 (48–87)
Sex, *n*	
Male	24
Female	35
Surgical history, *n*	
Cholecystectomy	5
Distal gastrectomy with Billroth I reconstruction	1
Purpose of EUS, *n*	
Close examination of lesions	52
Ampullary tumor	6
Pancreatic cyst	22
Pancreatic tumor	6
Biliary tumor	9
Biliary stone	9
Screening	7
Liver dysfunction	4
Bile duct dilation	2
Duodenal submucosal tumor	1
Echoendoscope used, *n*	
GF‐UE290	40
GF‐UE260‐AL5	13
GF‐UCT260	6
Antispasmodic agents, *n*	
Scopolamine butylbromide	21
Glucagon	38
Periampullary diverticula, *n*	8

The indications for EUS were screening in nine patients, including dilation of the bile duct (*n* = 2) and main pancreatic duct (*n* = 3) and close examination of lesions (*n* = 50), including six patients with ampullary tumors. Among these cases, periampullary diverticula were present in eight patients (14%).

Patient characteristics were also compared with regard to the degree of duodenal distention. In patients with excellent distention, the rates of surgical history and purpose of close examination of lesions were higher than that of patients with good or poor distention (Table S1).

### Outcomes

The grade of duodenal lumen distention with the GI method was excellent in 58% (34/59) cases, good in 34% (20/59), and poor in 7% (5/59, Table 3) of the analyzed cases.

**TABLE 3:** Results of endoscopic ultrasonography using the gel immersion method according to the grades of duodenal lumen distention

Effect of duodenal lumen distention	Overall (59 cases)	Excellent (34 cases, 58%)	Good (20 cases, 34%)	Poor (five cases, 7%)
Detection rate of the papilla	98% (58/59)	100% (34/34)	100% (20/20)	80% (4/5)
Detection rate of the intrapapillary pancreatobiliary ducts	78% (46/59)	91% (31/34)	65% (13/20)	40% (2/5)
Delineation rate of the papilla in the longitudinal view	66% (39/59)	71% (24/34)	65% (13/20)	40% (2/5)

The detection rate of the papilla was 98% (58/59), with one unsuccessful case due to poor duodenal lumen distention. The intrapapillary pancreatobiliary duct detection rate was 78% (46/59). According to the grades of duodenal lumen distention, the detection rates of excellent, good, and poor were 91% (31/34), 65% (13/20), and 40% (2/5), respectively. The delineation rate of the papilla in the longitudinal view was 66% (39/59). According to the grades of duodenal lumen distention, the delineation rates of excellent, good, and poor were 71% (24/34), 65% (13/20), and 40% (2/5), respectively.

In terms of differences in scope types, the successful delineation rates in the axial view were 98.1% (52/53) and 100% (6/6) for radial and convex EUS (*p* = 0.99), respectively, whereas the successful delineation rates in the longitudinal view were 64.1% (34/53) and 83.3% (5/6) for radial and convex EUS (*p* = 0.65), respectively.

The medians of the time required and gel volume used for the detection of the papilla were 3.1 (range, 1.0–13.4) min and 80 (range, 30–150) ml, respectively (Table 4). Those until delineation of the papilla in the longitudinal view were 7.9 (range, 1.9–28.6) min and 100 (range, 50–200) ml, respectively.

**TABLE 4:** Required volume of gel and required time for delineation

	**Median required volume of gel (ml)**	**Median required time (min)**
For detection of the papilla	80 (30–150)	3.1 (1.0–13.4)
For delineation of the papilla in the longitudinal view	100 (50–200)	7.9 (1.9–28.6)

The inter‐observer agreement for each outcome is shown in Table 5. An almost perfect agreement was obtained between the degree of duodenal lumen distention and papilla detection. There was substantial agreement in the detection of intrapapillary pancreatobiliary ducts and delineation of the papilla in the longitudinal view.

**TABLE 5:** Inter‐observer agreement (percent agreement measure and first‐order agreement [AC1] statistics) for each outcome

	**Percent agreement measure (%)**	**95% confidence interval**	**AC1 value**
Degree of duodenal lumen distended	75	0.81–0.94	0.87
Detection of the papilla	94	0.88–1.00	0.95
Detection of the intrapapillary pancreatobiliary ducts	78	0.48–0.86	0.67
Delineation of the papilla in the longitudinal view	85	0.55–0.90	0.73

Cutoff values in AC1 statistics: 0.00–0.20, slight; 0.21–0.40, fair; 0.41–0.60, moderate; 0.61–0.80, substantial; and 0.81–1.00, almost perfect agreement.

AC1, the first‐order agreement.

There were no adverse events associated with the GI method.

## DISCUSSION

Our findings indicate that GI‐EUS provides sufficient extension of the duodenal lumen with a small volume of gel, leading to a high detection rate of the duodenal papilla and a sufficient delineation rate of the papilla in the longitudinal view within a short time period and without adverse events. Considering that the usefulness of GI‐EUS for evaluation of the ampullary regions has not previously been well examined,^12^, ^13^ our study provides landmark data for the use of GI in pancreatobiliary EUS.

EUS is regarded as a crucial imaging modality in the pancreatobiliary field, and delineating the papilla and pancreatobiliary ducts is important for close examination of periampullary diseases,^1^, ^4^ for example, staging of ampullary tumors, detection of small biliary stones, pancreatobiliary maljunction, and pancreas division. However, the technical difficulty in the detection and examination of the papilla by EUS still exists. Kaneko et al. conducted a prospective comparative study between radial and convex EUS in 200 patients,^14^ and reported that the papilla was visualized in 41.4% of patients who underwent radial EUS and in 18.8% of those who underwent convex EUS. In a retrospective study conducted by Kanno et al. that included 3644 patients who underwent EUS surveillance,^15^ the pancreatobiliary junction was visualized in 80.0% of patients using radial EUS and in 89.5% of those using convex EUS. The large difference in the visualization rate of the papilla between these two reports is presumably caused by the difference in the definition of the visualization of the papilla. In Kaneko et al.’s study, the successful visualization of the ampullary region was defined as the penetration of the muscularis propria by both the common bile duct and pancreatic duct as visualized on a single image. In contrast, according to Kanno et al.’s study, the papilla was considered well observed when the distal ends of both the bile duct and main pancreatic ducts were clearly observed with the awareness of the positional relation between these ducts, the duodenal ampulla, and the duodenal muscular layer.

In this study, the detection of the papilla was considered successful in cases with visualization of a protruded region in the duodenum, where the pancreatobiliary duct penetrated the duodenal muscular layer. This definition was similar to Kanno et al.’s definition. In our study, the detection rate of papilla was 98%, which might be higher than that reported by Kanno et al. This difference might be caused by the effect of the GI method. In other words, a gel that distends the duodenal lumen might help make the recognition of the papilla easier.

In addition, the current study defined successful delineation of the papilla in the longitudinal view as the simultaneous visualization of the papilla and pancreatobiliary duct penetrating the duodenal muscular layer in the longitudinal view. This definition was similar to that of Kaneko et al. However, the successful delineation rate of the bile and pancreatic ducts was 66%, which was higher than that reported by Kaneko et al. This difference was speculated to be due to the strict definition of papilla delineation in Kaneko et al.’s study. Our study allowed the confirmation of the bile duct, pancreatic duct, and papilla in the longitudinal view with a series of images, and was not concerned with the delineation of the pancreatic duct, bile duct, and papilla in a single image.

In our study, the delineation rate of the papilla in the longitudinal view was lower than that of detection of the papilla alone, suggesting that the successful longitudinal delineation of the papilla is still challenging even though the GI method provides sufficient lumen distention. Although the detection rates of the intrapapillary pancreatobiliary ducts were different according to the degree of the lumen distention, the delineation rate of the papilla in the longitudinal view among the cases with excellent distention was almost the same as that among the cases with good distention. This indicated that the degree of the lumen distention did not significantly influence the successful delineation of the papilla in a longitudinal view. Therefore, the successful delineation of the pancreatobiliary ducts in the longitudinal view requires several factors other than lumen distention of the duodenal lumen, such as technical proficiency or anatomical situation, as well as sufficient distention of the duodenal lumen over a sufficient time period.

The volume of the gel used for the detection and delineation of the papilla in the longitudinal view was relatively low. Thus, GI‐EUS can provide sufficient distention of the duodenal lumen with a small amount of liquid. Although the amount of gel and water for the detection and delineation of the papilla in a longitudinal view should be compared, this is a conclusive advantage, especially for patients who need to avoid the use of large volumes, such as those with cardiac or renal dysfunction.

The disadvantage of VISCOCLEAR is the cost; 200 ml of VISCOCLEAR costs 2000 JPN (approximately $15, based on current conversion rates). Economic considerations may limit the use of GI‐EUS. Therefore, GI‐EUS should be considered in cases that require close examination in the periampullary region or in cases with the difficulty of filling liquid in the duodenal lumen, such as evaluation of the ampullary tumor, dilation of the pancreatobiliary duct with unknown cause, as well as patients with periampullary diverticula.

In this study, we used AC1 statistics, which have recently attracted attention, to evaluate the inter‐observer agreement.^16^ Although the kappa statistic is widely accepted as an index of reproducibility, it has been reported to be vulnerable to the prevalence and presence of bias.^17^ All values obtained in this study were high, and small negative results may be improperly exaggerated. Thus, we used the AC1 statistics as an alternative robust statistic.

The limitations of our study should be acknowledged. First, this was a retrospective, single‐institution study, and, therefore, selection bias cannot be ruled out. Therefore, a prospective multicenter randomized controlled study of a large number of patients is needed to confirm the reliability of our results. Second, we used both radial and convex linear‐arrayed echoendoscopes for GI‐EUS; it is possible that the detection ability could differ between the two echoendoscope types which might influence our findings. Lastly, this study aimed to verify the papilla detection and delineation ability of GI‐EUS; thus, the diagnostic ability was not investigated. The diagnostic ability of GI‐EUS for periampullary lesions will be investigated in a future study.

In conclusion, the GI method provided sufficient duodenal lumen distention, leading to high rates of detection and delineation of the papilla in a short time, with a small amount of gel used. Although a further randomized controlled trial that compares water and GI‐EUS for the evaluation of the papilla is needed to verify the results, our study indicated that GI‐EUS is a safe and efficacious method for examining the peri‐ampullar region.

## CONFLICT OF INTEREST

Akio Katanuma received honoraria for lecture fees from Olympus Co., Tokyo, Japan. The other authors have no conflict of interest to declare.

## FUNDING INFORMATION

None

## Supporting information



Supplementary Information

**Table S1**. Patients' characteristics based on the degree of duodenal distention (n=59)
