# Supplementary material for: Efficacy of gel immersion endoscopic ultrasonography for delineating the duodenal papilla and pancreatobiliary ducts: A retrospective study with video
Source: DEN Open. 2022 Aug 4;3(1):e158. doi: 10.1002/deo2.158 (PMC9353120; doi:10.1002/deo2.158)
Supplement: Supplementary file 2 — Table S1. Patients' characteristics based on the degree of duodenal distention (n=59) [file DEO2-3-e158-s001.docx]

| **Supplemental Table 1.** Patients’ characteristics based on the degree of duodenal distention (n=59) | | | |
| --- | --- | --- | --- |
| Degree of duodenal distention | Excellent  (34 cases) | Good or Poor  (25 cases) | P value |
| Age, median (range), years | 68 (49-87) | 69 (48-87) | 0.818 |
| Sex, n |  |  | 0.435 |
| Male | 16 (47.1%) | 9 (36.0) |  |
| Female | 18 (52.9%) | 16 (64.0) |  |
| Surgical history, n | 6 (17.6%) | 0 (0%) | 0.034 |
| Purpose of EUS, n |  |  | 0.018 |
| Close examination of lesions | 22 (64.7%) | 8 (32.0%) | 52 |
| Screening | 12 (35.3%) | 17 (68.0%) | 7 |
| Echoendoscope used, n |  |  | 1 |
| Radial type | 30 (88.2%) | 23 (92.0%) |  |
| Convex type | 4 (11.8%) | 2 (8.0%) |  |
| Antispasmodic agents, n |  |  | 1 |
| Scopolamine butylbromide | 12 (35.3%) | 9 (36.0%) |  |
| Glucagon | 22 (64.7%) | 16 (64.0%) |  |
| Periampullary diverticula, n | 6 (17.6%) | 2(8.0%) | 0.477 |
